# Antifungal Drugs and Drug-Induced Liver Injury: A Real-World Study Leveraging the FDA Adverse Event Reporting System Database

**DOI:** 10.3389/fphar.2022.891336

**Published:** 2022-04-28

**Authors:** Zhi-Xuan Zhou, Xue-Dong Yin, Yu Zhang, Qi-Hui Shao, Xin-Yu Mao, Wen-Juan Hu, Yun-Lin Shen, Bin Zhao, Zhi-Ling Li

**Affiliations:** ^1^ Department of Pharmacy, Shanghai Children’s Hospital, Shanghai Jiao Tong University, Shanghai, China; ^2^ Shanghai Jiao Tong University School of Medicine, Shanghai, China; ^3^ Department of Pharmacy, Nanyang Central Hospital, Affiliated Hospital of Zhengzhou University, Nanyang, China; ^4^ Department of Neonatology, Shanghai Children’s Hospital, Shanghai Jiao Tong University, Shanghai, China; ^5^ Pharmacy Department, Peking Union Medical College Hospital, Peking Union Medical College, Chinese Academy of Medical Sciences, Beijing, China

**Keywords:** DILI, antifungal drugs, pharmacovigilance, adverse event reporting system, epidemiology

## Abstract

**Aims:** We aimed to estimate the risk of drug-induced liver injury (DILI) from various antifungal treatments with azoles and echinocandins causing in real-world practice.

**Methods:** We performed disproportionality and Bayesian analyses based on data from the first quarter in 2004 to the third quarter in 2021 in the Food and Drug Administration Adverse Event Reporting System to characterize the signal differences of antifungal drugs-related DILI. We also compared the onset time and mortality differences of different antifungal agents.

**Results:** A total of 2943 antifungal drugs-related DILI were identified. Affected patients tended to be aged >45 years (51.38%), with more males than females (49.03% vs. 38.09%). Antifungal drug-induced liver injury is most commonly reported with voriconazole (32.45%), fluconazole (19.37%), and itraconazole (14.51%). Almost all antifungal drugs were shown to be associated with DILI under disproportionality and Bayesian analyses. The intraclass analysis of correlation between different antifungal agents and DILI showed the following ranking: caspofungin (ROR = 6.12; 95%CI: 5.36–6.98) > anidulafungin (5.15; 3.69–7.18) > itraconazole (5.06; 4.58–5.60) > voriconazole (4.58; 4.29–4.90) > micafungin (4.53; 3.89–5.27) > posaconazole (3.99; 3.47–4.59) > fluconazole (3.19; 2.93–3.47) > ketoconazole (2.28; 1.96–2.64). The onset time of DILI was significantly different among different antifungal drugs (*p* < 0.0001), and anidulafungin result in the highest mortality rate (50.00%), while ketoconazole has the lowest mortality rate (9.60%).

**Conclusion:** Based on the Food and Drug Administration Adverse Event Reporting System database, antifungal drugs are significantly associated with DILI, and itraconazole and voriconazole had the greatest risk of liver injury. Due to indication bias, more clinical studies are needed to confirm the safety of echinocandins.

## Introduction

Drug-induced liver injury (DILI) is a common and serious adverse drug reaction, defined as liver damage caused by a drug or herbal product resulting in abnormal liver tests or liver dysfunction, after reasonable exclusion of competing etiologies (Raschi E et al., 2014). Antifungal drugs can be classified as polyenes, antimetabolite—flucytosine (5-FC), azoles, echinocandins, the latter two being more common, which are the first-line option for the prevention and treatment of fungal infections caused by immunosuppression (Nett and Andes, 2016). In recent years, due to the epidemic of acquired immunodeficiency syndrome (AIDS) and the advancement of immunosuppressive techniques, the number of patients with severely weakened immune systems has increased, and the incidence of fungal infections has continued to rise (Tverdek et al., 2016). With the wide application of antifungal drugs, the safety of antifungal drugs has been concerned.

There are many adverse reactions to antifungal drugs, such as hepatotoxicity and hormone-related effects (gynecomastia, alopecia, decreased libido, oligospermia, azoospermia and so on), among which hepatotoxicity is the most common. An article summarized that all azoles have abnormal liver function and hepatotoxicity, and the frequency of adverse reactions varies by drug and patient population (Benitez and Carver, 2019). A real-world study found that about 2.9% of all reported drug-induced liver injuries are associated with antifungal drugs (Raschi et al., 2014). Another retrospective study reported the prevalence of micafungin-associated DILI was 10.6% (Mullins et al., 2020). It can be seen that almost all antifungal drugs have certain hepatotoxicity.

However, the current studies are based on a case or retrospective study of a drug, and there are few real-world studies. The existing real-world study was in 2014, and the data need to be further updated. In this context, this study aims to characterize the liver injury induced by various antifungal drugs in a large population by using FAERS. We further examined and compared the onset-time and outcomes of liver injury with different antifungal drugs.

## Methods

### Data Source

**FIGURE 1:**
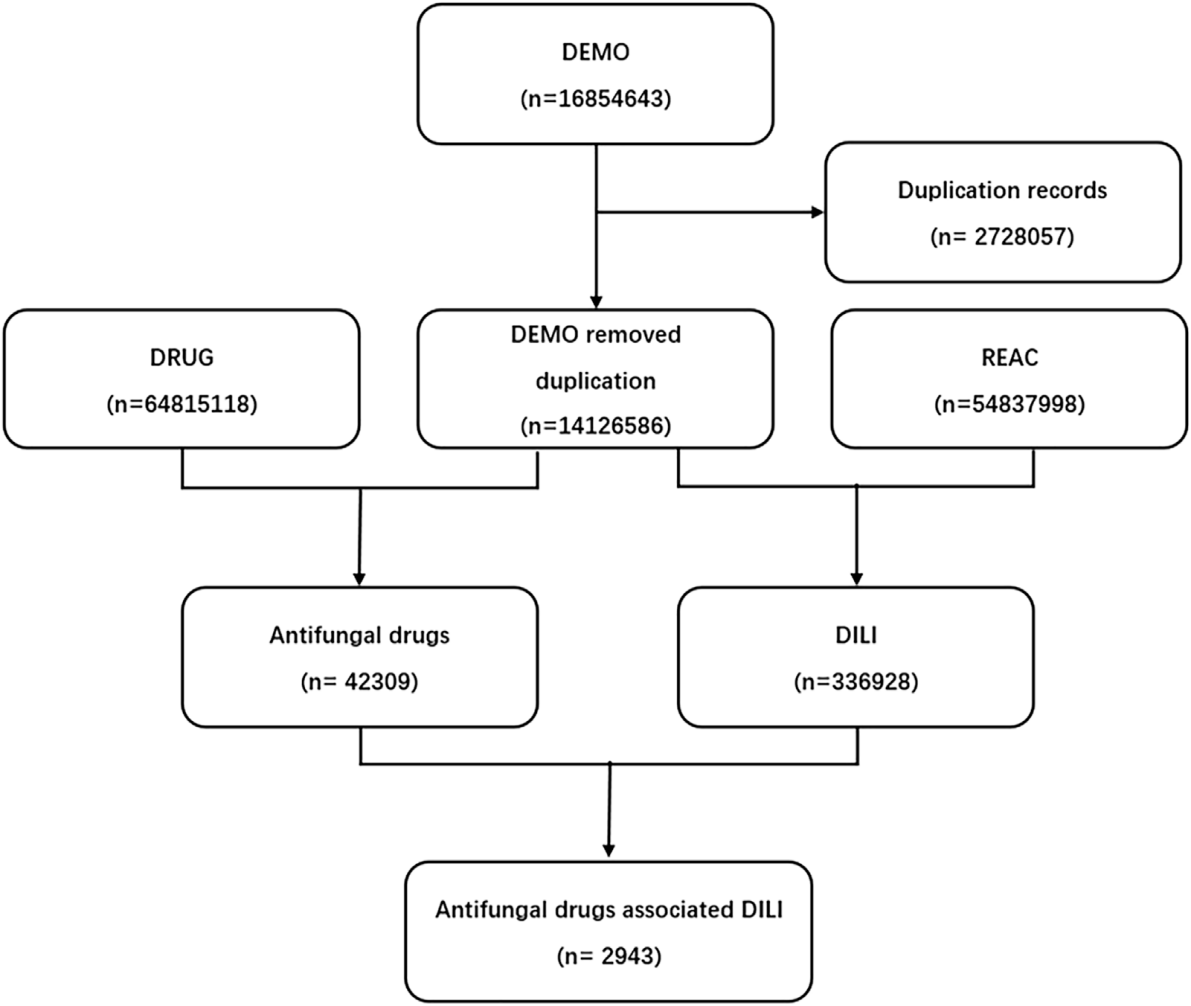
Process of the selection of cases of antifungal drugs-associated liver injury from the Food and Drug Administration’s Adverse Event Reporting System database. DEMO, demographic information; DRUG, drug information; REAC, adverse events.

We conducted a retrospective pharmacovigilance study using the FAERS database from the first quarter of 2004 to the third quarter of 2021. FDA adverse event reporting system (FAERS) is a database designed to support FDA’s post-marketing monitoring plan for drugs and therapeutic biological products, which includes all Adverse drug reaction (ADR) signals and medication error information collected by FDA. A FAERS data contains demographic information, drug information, adverse events, patient outcomes, indications, duration of use, time to adverse reactions, and more. Finally, a total of 16854643 reports were obtained from the FAERS database (Figure 1).

### Adverse Event and Drug Identification

DILI cases were obtained by searching using the Medical Dictionary for Regulatory Activities (MedDRA) (version 23.0), and the preferred terms are shown in Table 1. Study drugs were antifungal triazoles (ketoconazole, miconazole, clotrimazole, fluconazole, voriconazole, itraconazole, isavuconazole, posaconazole) and echinocandins (caspofungin, micafungin, anidulafungin) on the market.

**TABLE 1:** MedDRA preferred terms used to retrieve liver events in FAERS.

PT
Liver injury
Liver damage
Liver necrosis
Hepatic damage
Hepatotoxicity
Hepatopathy
Hepatic disease
Hepatitis
Nonalcoholic fatty liver disease
Liver fatty infiltration
Steatohepatitis
Hepatic steatosis
Jaundice
Icterus
Cholestasis
Bile duct damage
Biliary cholangitis
Hepatobiliary disease
Hepatic encephalopathy
Hepatic failure
Hepatic vascular injury
Hepatic cirrhosis
Portal hypertension
DILI
Hepatic necrosis
Hepatocellular injury
Hepatomegaly
Hepatic enzyme abnormal
Hepatic enzyme increased
Transaminases increased
Transaminases abnormal
Blood bilirubin abnormal
Blood bilirubin increased
Aspartate aminotransferase abnormal
Aspartate aminotransferase increased
Hepatic injury
Hepatic function abnormal
Hepatocellular damage
Cirrhosis
Hyperbilirubinaemia
Liver transplant
Alanine aminotransferase abnormal
Alanine aminotransferase increased
Ammonia increased
Bilirubin conjugated increased
Bilirubin urine
Blood bilirubin unconjugated
Increased
Coma hepatic
Hyperammonaemia
Liver function test abnormal
Mixed hepatocellular-cholestatic injury
Urine bilirubin increased

PT, preferred terms.

### Data Mining

Based on the principles of Bayesian analysis and disproportionality analysis, we used the reporting odds ratio (ROR), the proportional reporting ratio (PRR), the Bayesian confidence propagation neural network and the multi-item gamma Poisson shrinker algorithms to explore the associations between antifungal drugs and DILI (Evans et al., 2001; Szarfman et al., 2002; van Puijenbroek et al., 2002; Hauben et al., 2005; Norén et al., 2006; Ooba and Kubota, 2010; Szumilas, 2010). A two-by-two contingency table (Table 2) of reported event counts for specific drug and other drugs was constructed to calculate ROR, PRR, information component (IC), and empirical Bayesian geometric mean (EBGM). The former two belong to disproportionality analysis, while the latter two belong to Bayesian analysis. The calculation formula and criteria follow: Table 2.

**TABLE 2:** Two-by-two contingency table for disproportional analysis.

	DILI	All other adverse drug reactions	Total
Antifungal drugs	a	c	a + c
All other drugs	b	d	b + d
Total	a + b	c + d	a+b + c + d

ROR = (a/b)/(c/d),  95% CI=eln(ROR)±1.96(1/a+1/b +1/c+1/d)^0.5(criteria: 95% CI>1, n≥2) ;

PRR = (a / [a + c]) / (b / [b + d]), χ2=Σ([O−E]^2/E),(O=a, E=[a+b][a+c]/[a+b+c+d])(criteria:PRR≥2,χ2≥4, n≥3) ; IC=log⁡2a*(a+b+c+d)/([a+c][a+b]), IC025=eln(IC)−1.96(1/a+1/b+1/c+1/d)^0.5(criteria:IC025>0) ;

EBGM=a*(a+b+c+d)/([a+c][a+b]), EBGM05=eln(EBGM)−1.64(1/a+1/b+1/c+1/d)^0.5(criteria:EBGM05≥2, n>0) .

CI, indicates confidence interval; n, indicates the number of co-occurrences; *χ*2, indicates chi-squared; IC, indicates information component; IC025, indicates the lower limit of the 95% two-sided CI of the IC; EBGM05, the lower limit of the 90% one-sided CI of the EBGM.

### Statistical Analysis

Descriptive analyses were used to summarize the characteristics of adverse event reports on antifungal drug-related liver injuries collected from the FAERS database. We analyzed the age, sex, reporters, country, area and reporting time distribution of different antifungal agents, and compared the onset time and mortality differences of different antifungal agents.

## Results

### Descriptive Analysis

**FIGURE 2:**
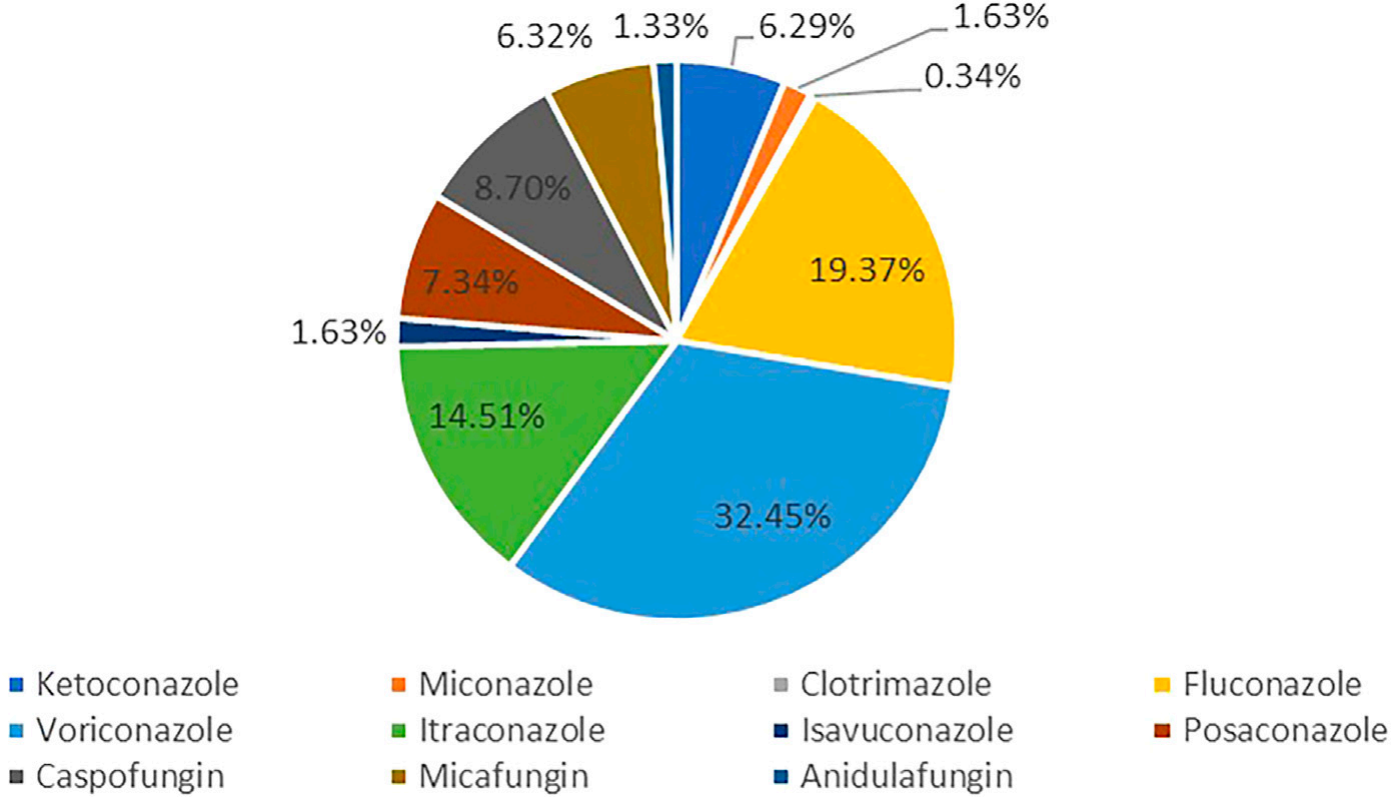
Proportion of antifungal drugs-related liver injury.

FAERS database from the first quarter in 2004 to the third quarter in 2021 contained 42309 antifungal drugs-related adverse events and 336928 DILI-related reports, among these 2943 were reported for DILI after using antifungal (Figure 1). The clinical characteristics of patients with antifungal drug-induced liver injuries were described in Table 3 and Figure 2. Most of the patients were older than 45 years (51.38%), and men accounted for a larger proportion than women in all reports (49.03% vs. 38.09%). Most cases were reported from Europe (40.88%), Asia (25.35%) and North America (23.41%), and were reported by the physician (40.47%). More and more cases were reported from 2016 (5.27%) to 2020 (9.14%), reflecting the significantly increased usage of antifungal drugs in recent years. Voriconazole ranked first in the number of cases (955), followed by fluconazole and itraconazole.

**TABLE 3:** Clinical characteristics of patients with antifungal drugs-associated DILI sourced from the FDA Adverse Event Reporting System database (2004q1 to 2021q3).

Characteristics	Reports, n (%)
Patient age (year)	
<18	218(7.41%)
18–44	511 (17.36%)
45–64	755(25.65%)
65–74	432 (14.68%)
75–84	264 (8.97%)
≥85	61 (2.07%)
Unknow	702(23.85%)
Reporter	
Consumer	306 (10.40%)
Lawyer	1 (0.03%)
Other health-professional	808(27.45%)
Pharmacist	345 (11.72%)
Physician	1191(40.47%)
Unknow	292 (9.92%)
Patient gender	
Female	1121 (38.09%)
Male	1443(49.03%)
Unknow	379(12.88%)
Year	
2004	136 (4.62%)
2005	132 (4.49%)
2006	134 (4.55%)
2007	120 (4.08%)
2008	117(3.98%)
2009	137 (4.66%)
2010	152 (5.16%)
2011	129 (4.38%)
2012	154 (5.23%)
2013	156(5.30%)
2014	124 (4.21%)
2015	174 (5.91%)
2016	155 (5.27%)
2017	183 (6.22%)
2018	231(7.85%)
2019	262 (8.90%)
2020	269 (9.14%)
2021	171 (5.81%)
Unknow	6 (0.20%)
Area	
Africa	26 (0.88%)
Asian	746(25.35%)
Europe	1203(40.88%)
North America	689 (23.41%)
Oceania	39 (1.33%)
South America	34 (1.16%)
Unknow	206 (7.00%)
Antifungal drugs	
Ketoconazole	188 (6.29%)
Miconazole	48 (1.63%)
Clotrimazole	10 (0.34%)
Fluconazole	570 (19.37%)
Voriconazole	955 (32.45%)
Itraconazole	427 (14.51%)
Isavuconazole	48 (1.63%)
Posaconazole	216 (7.34%)
Caspofungin	256 (8.70%)
Micafungin	186 (6.32%)
Anidulafungin	39 (1.33%)

### Disproportionality Analysis and Bayesian Analysis

Data showed a strong association between antifungals and DILI, with no positive signals detected only for miconazole and clotrimazole. In addition, there were two other drugs with one or two negative signals according to the criteria of the four algorithms: which are ketoconazole in EBGM: 2.21(1.95), and isavuconazole in ROR: 1.22(0.92–1.63), PRR: 1.22(1.88) and EBGM: 1.22(0.96). The intraclass analysis of correlation between different antifungal agents and DILI showed the following ranking: caspofungin (ROR = 6.12; 95%CI: 5.36–6.98) > anidulafungin (5.15; 3.69–7.18) > itraconazole (5.06; 4.58–5.60) > voriconazole (4.58; 4.29–4.90) > micafungin (4.53; 3.89–5.27) > posaconazole (3.99; 3.47–4.59) > fluconazole (3.19; 2.93–3.47) > ketoconazole (2.28; 1.96–2.64) (Table 4).

**TABLE 4:** Association of antifungal drugs with DILI.

Drugs	N	ROR	PRR	IC	EBGM
		(95% Two-sided CI)	(χ2)	(IC025)	(EBGM05)
Ketoconazole	188	2.28 (1.96, 2.64)*	2.21 (127.22)*	1.14 (0.99)*	2.21(1.95)
Miconazole	48	0.30 (0.23, 0.40)	0.31 (77.37)	−1.71()	0.31 (0.24)
Clotrimazole	10	0.16 (0.08, 0.29)	0.16 (44.96)	−2.64 ()	0.16 (0.10)
Fluconazole	570	3.19 (2.93, 3.47)*	3.03 (793.68)*	1.60 (1.47)*	3.03 (2.82)*
Voriconazole	955	4.58 (4.29, 4.90)*	4.22 (2400.98)*	2.08 (1.94)*	4.22 (3.99)*
Itraconazole	427	5.06 (4.58, 5.60)*	4.62 (1237.69)*	2.21 (1.99)*	4.61 (4.24)*
Isavuconazole	48	1.22 (0.92, 1.63)	1.22 (1.88)	0.28 (0.21)*	1.22 (0.96)
Posaconazole	216	3.99 (3.47, 4.59)*	3.72 (440.63)*	1.90 (1.65)*	3.72 (3.31)*
Caspofungin	256	6.12 (5.36, 6.98)*	5.45 (952.64)*	2.45 (2.14)*	5.45 (4.88)*
Micafungin	186	4.53 (3.89, 5.27)*	4.18 (460.10)*	2.06 (1.77)*	4.17 (3.68)*
Anidulafungin	39	5.15 (3.69, 7.18)*	4.69 (115.82)*	2.23 (1.60)*	4.69 (3.55)*

ROR, reporting odds ratio; CI, confidence interval; PRR, proportional reporting ratio; χ^2^, chi-squared; IC, information component; EBGM, empirical Bayesian geometric mean; “*” suggests that antifungal agents are associated with DILI.

### Onset Times of DILI

The median onset times of DILI for each antifungal drugs are summarized in Table 5. The onset time of DILI was significantly different among different antifungal drugs (*p* < 0.0001). Significant differences were noted for ketoconazole vs. posaconazole (*p* = 0.0460), ketoconazole vs. caspofungin (*p* = 0.0006), ketoconazole vs. micafungin (*p* = 0.0018), voriconazole vs. caspofungin (*p* = 0.0248), itraconazole vs. caspofungin (*p* < 0.0001), itraconazole vs. micafungin (*p* = 0.0005).

**TABLE 5:** Onset times of DILI associated with antifungals.

Antifungal drugs	M(IQR)(d)
Ketoconazole	21 (7–40)
Miconazole	22 (2.5–45.75)
Fluconazole	8 (3–17.25)
Voriconazole	8 (2–20)
Itraconazole	11 (4–32.5)
Isavuconazole	7 (0–56.5)
Posaconazole	6 (2–19)
Caspofungin	5 (2–11)
Micafungin	5 (1.5–12.5)
Anidulafungin	4 (1–12)

M, median; IQR, interquartile range; d, days.

### Outcomes due to DILI

**FIGURE 3:**
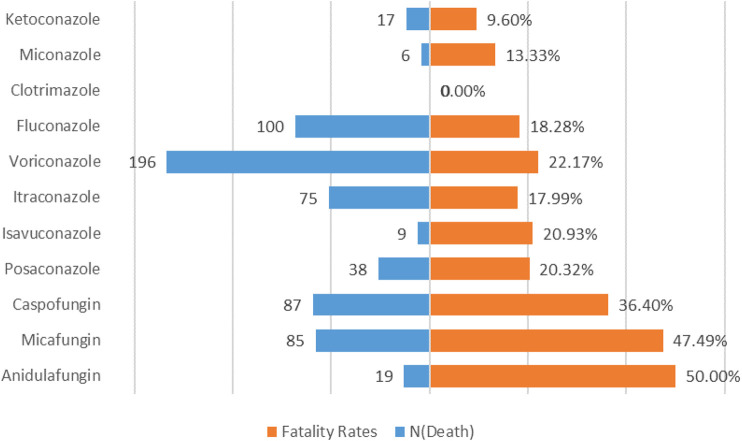
Mortality rate for DILI associated with antifungal drugs.

To analyze the prognosis of antifungal drugs-induced liver injuries, we calculated the proportion of outcomes (death, disability, hospitalization, life-threatening, other serious and required intervention) due to DILI after various antifungal drugs treatments, and the results are shown in Table 6. Patients with antifungal-related liver damage tended to have poor outcomes, with approximately 42.21% of patients hospitalized and 22.86% dying. In addition, a significant difference in the mortality rate of DILI was found between different antifungal drugs (*p* < 0.0001). Anidulafungin results in the highest mortality rate (50.00%), while ketoconazole has the lowest mortality rate (9.60%). The mortality rate for each drug is shown in Figure 3.

**TABLE 6:** Outcomes events of DILI.

	Ketoconazole	Miconazole	Clotrimazole	Fluconazole	Voriconazole	Itraconazole	Isavuconazole	Posaconazole	Caspofungin	Micafungin	Anidulafungin
Congenital Anomaly	0(0.00)	1(2.22)	0(0.00)	0(0.00)	0(0.00)	2(0.48)	0(0.00)	0(0.00)	0(0.00)	0(0.00)	0(0.00)
Death	17(9.60)	6(13.33)	0(0.00)	100(18.28)	196(22.17)	75(17.99)	9(20.93)	38(20.32)	87(36.40)	85(47.49)	19(50.00)
Disability	3(1.69)	1(2.22)	0(0.00)	10(1.83)	24(2.71)	9(2.16)	0(0.00)	7(3.74)	8(3.35)	9(5.03)	1(2.63)
Hospitalization - Initial or Prolonged	67(37.85)	24(53.33)	6(66.67)	292(53.38)	322(36.43)	157(37.65)	17(39.53)	88(47.06)	123(51.46)	57(31.84)	14(36.84)
Life-Threatening	11(6.21)	3(6.67)	0(0.00)	51(9.32)	86(9.73)	22(5.28)	0(0.00)	23(12.30)	41(17.15)	31(17.32)	6(15.79)
Other Serious	117(66.10)	28(62.22)	7(77.78)	327(59.78)	638(72.17)	265(63.55)	39(90.70)	132(70.59)	126(52.72)	101(56.42)	21(55.26)
Required Intervention to Prevent Permanent Impairment/Damage	3(1.69)	0(0.00)	0(0.00)	4(0.73)	6(0.68)	2(0.48)	0(0.00)	2(1.07)	1(0.42)	0(0.00)	0(0.00)

## Discussion

Drug-induced liver injury is classified as intrinsic, idiosyncratic and indirect, and the idiosyncratic type can be divided into hepatocellular injury, cholestatic liver injury and mixed liver injury (Garcia-Cortes et al., 2020). Patients with alanine aminotransferase (ALT) > 5 times the upper limit of normal or alkaline phosphatase (ALP) > 2 times the upper limit of normal were considered suspected DILI. If ALT/ALP ≥5, it is defined as Hepatocellular injury; if ALT/ALP ≤2, it is defined as Cholestatic liver injury; if 2 < ALT/ALP <5, it is defined as Mixed liver injury (Danan and Theschke, 2015).

Triazole drugs prevent the synthesis of ergosterol by inhibiting C14 (a sterol demethylase), thereby reducing sterol precursors and ergosterol, and destroying the integrity of the fungal cell membrane, thus achieving the antifungal effect (Nett and Andes, 2016). The exact mechanism of hepatotoxicity induced by triazole drugs remains unclear. They competitively inhibit liver oxidative metabolism by rapidly and reversibly binding CYP450 metabolic enzymes, and are both substrates and inhibitors of various CYP450 metabolic enzymes, with potential for drug interactions (Zonios and Bennett, 2008). Drug interactions increase the risk of increased toxicity leading to liver damage. Itraconazole is metabolized primarily by the CYP450 isoenzyme CYP3A4; voriconazole is metabolized by the CYP450 isoenzymes CYP2C9, CYP2C19, and CYP3A4 (Haria et al., 1996; Alffenaar et al., 2009). In addition, itraconazole, but not voriconazole, is an inhibitor of gastric P-glycoprotein, a transmembrane efflux pump that limits blood drug concentrations by expelling the drug into the intestinal lumen (Wang et al., 2002). Itraconazole inhibits drug efflux by inhibiting P-glycoprotein, resulting in increased plasma concentrations and increased systemic exposure of the drug, so itraconazole has a higher risk of liver damage. Fluconazole is metabolized mainly through the kidney (Shiba et al., 1990), but not extensively through the liver, so its hepatotoxicity is relatively low. The echinocandins damage fungal cell walls by inhibiting the synthesis of B-1,3 glucan, a fungal cell wall polysaccharide essential to many fungi. The echinocandins are eliminated mainly by non-enzymatic degradation to an inactive product. Although they are not significantly metabolized by CYP450 enzymes, caspofungin and micafungin are metabolized in the liver and thus have less hepatotoxicity (Nett and Andes, 2016).

This study found that almost all antifungal drugs can cause liver damage, and the association of echinocandins is significantly higher than that of triazoles. In previous studies, hepatotoxicity of echinocandins was considered to be significantly lower than that of triazoles (Tverdek et al., 2016; Kyriakidis et al., 2017). An *in vitro* study found that caspofungin exhibited mild hepatotoxicity, whereas fluconazole and voriconazole exhibited higher hepatotoxicity (Doß et al., 2017). Due to the low hepatotoxicity of echinocandins, some echinocandins have been used safely in patients with pre-existing liver damage, namely caspofungin in patients with chronic liver disease and post-liver transplantation (Mycamine., 2010). A retrospective study found that patients with abnormal liver enzymes at baseline had an increased overall incidence of liver injury compared with patients without elevated liver enzymes at baseline (Takeda et al., 2007). Since echinocandins are widely used in patients with liver damage themselves, if the liver damage worsens after medication, the case may be reported to FAERS as DILI. Therefore, patients with echinocandins-related liver damage will be higher than the actual liver damage caused by drugs. The basic number of patients is large, so there is a strong correlation between echinocandins and liver damage, which is also a shortcoming of this study. Due to indication bias in real-world studies, the ROR of echinocandins was higher than that of triazoles.

Of note, we also found that the correlation between different triazoles and DILI showed the following ranking: itraconazole > voriconazole > fluconazole > ketoconazole. Previous studies considered ketoconazole to be the most hepatotoxic triazole, and ketoconazole was withdrawn from the market precisely due to its hepatotoxic effect and better-evaluated alternatives, so the ROR of this study was reduced accordingly. Numerous studies have shown that voriconazole and itraconazole are more hepatotoxic than other triazoles (Kullberg et al., 2005; Wang et al., 2010), possibly due to their ability to inhibit CYP450, resulting in significant drug-drug interactions, which in turn alter circulating plasma levels of concomitant drugs and increase liver toxicity.

Another finding is that the mortality rate of echinocandins was significantly higher than that of triazole. Among echinocandins, anidulafungin has the highest mortality rate. Anidulafungin is the only echinocandin that is not metabolized through the liver, and dose adjustment is not required even in patients with severe hepatic insufficiency (Patil and Majumdar, 2017), therefore, clinicians may prefer to use anidulafungin in patients with a history of hepatotoxicity. A small retrospective study found that anidulafungin was used more than micafungin in patients with liver failure, confirming real-world channel bias (van der Geest et al., 2016). Another retrospective study also found that baseline liver function impairment and other more serious comorbidities were more likely to be in patients using anidulafungin (van der Geest et al., 2016; Vekeman et al., 2018). Therefore, the highest mortality rate of anidulafungin may be affected by the patients’ condition. However, due to the significant risk of liver injury of azole drugs, clinicians will strictly grasp the indications and monitor the level of liver function when using them and stop taking them in time to relieve the condition when liver enzymes are significantly increased. In most cases using triazoles, liver enzymes returned to normal and symptoms disappeared within a few weeks after drug withdrawal (Song and Deresinski, 2005), so triazole mortality is relatively low.

We acknowledge that our research has certain limitations. First, the FAERS database is a fully open website, so the absolute authenticity of data cannot be guaranteed, and there may be duplicate samples and imperfect data. Due to underdeveloped information in some regions, a lot of data have not been recorded in the database, for example, the sample of Africa is only 0.88%. Secondly, this study selected all patients with liver damage after medication, which could not exclude further aggravation of liver damage in patients with abnormal liver function, so there was a certain bias. Finally, this paper did not use Roussel Uclaf Causality Assessment Method (RUCAM**)** to define DILI, but screened all patients with liver enzyme abnormalities or liver damage, so the data were not accurate to some extent. Therefore, the FAERS database cannot be directly used to calculate the incidence of DILI, but it can be used as a pharmacovigilance tool to remind pharmacists to use antifungals with caution.

The current study showed that antifungal drugs are significantly associated with DILI, and itraconazole and voriconazole had the greatest risk of liver injury. Clinicians are advised to monitor and consider patients’ liver function when taking antifungal drugs. At the same time, due to indication bias, more clinical studies are needed to confirm the safety of echinocandins.

## Data Availability

The original contributions presented in the study are included in the article/Supplementary Material, further inquiries can be directed to the corresponding authors.
